# New Guar Biopolymer Silver Nanocomposites for Wound Healing Applications

**DOI:** 10.1155/2013/912458

**Published:** 2013-09-23

**Authors:** Runa Ghosh Auddy, Md Farooque Abdullah, Suvadra Das, Partha Roy, Sriparna Datta, Arup Mukherjee

**Affiliations:** Department of Chemical Technology, University of Calcutta, 92 A.P.C. Road, Kolkata, West Bengal 700009, India

## Abstract

Wound healing is an innate physiological response that helps restore cellular and anatomic continuity of a tissue. Selective biodegradable and biocompatible polymer materials have provided useful scaffolds for wound healing and assisted cellular messaging. In the present study, guar gum, a polymeric galactomannan, was intrinsically modified to a new cationic biopolymer guar gum alkylamine (GGAA) for wound healing applications. Biologically synthesized silver nanoparticles (Agnp) were further impregnated in GGAA for extended evaluations in punch wound models in rodents. SEM studies showed silver nanoparticles well dispersed in the new guar matrix with a particle size of ~18 nm. In wound healing experiments, faster healing and improved cosmetic appearance were observed in the new nanobiomaterial treated group compared to commercially available silver alginate cream. The total protein, DNA, and hydroxyproline contents of the wound tissues were also significantly higher in the treated group as compared with the silver alginate cream (*P* < 0.05). Silver nanoparticles exerted positive effects because of their antimicrobial properties. The nanobiomaterial was observed to promote wound closure by inducing proliferation and migration of the keratinocytes at the wound site. The derivatized guar gum matrix additionally provided a hydrated surface necessary for cell proliferation.

## 1. Introduction

Wounds inflict disruption of the cellular and anatomic continuity of tissues and thus affect physiological functions. Wound healing therefore is a complex and important biological response that helps in the restoration of tissue integrity and body functions [1]. The process of wound healing involves a highly integrated cascade of continuous and overlapping biological events. Cellular discontinuity and appearance of nascent cells also extend susceptible regions for microbial infections. Coordinated completion of a series of biochemical events and protection against invading organisms are therefore necessary for tissue rebuilding, haemostasis, and maturation [2]. The principle objective in wound management is to heal the wound in the shortest possible time, with minimal pain, discomfort, and scarring to the patient, and must occur in a physiological environment conducive to tissue repair and regeneration [3]. Delayed healing often results in bacterial infections, stress, and nutritional deficiencies [4]. Currently, the demand for new wound healing materials with inherent antimicrobial properties is on the rise. Polysaccharides associate low antigenicity and are often a choice for wound management scaffolds [5, 6]. Chitosan and different starch derivatives were experimented earlier as wound healing materials. Polysaccharides are excellent growth scaffolds, but their wound healing application is limited as they are vulnerable to microbial contaminations. Chitosan however is one exception. The cationic surface of chitosan in effect provides a reasonable surface sterility for tissue regeneration. This work therefore concentrates on cationic modification of polysaccharide end groups so that new material surfaces can be developed for facilitated tissue regeneration during wound management. Guar gum is a nontoxic biopolymer derived from the seeds of *Cyamopsis tetragonoloba*. Because of its high biocompatibility and biodegradability, it is used extensively as a biomaterial in a plethora of biological and technological processes [7]. Guar and xanthan gums were patented earlier as bioabsorbable materials for wound dressing [8]. Guar gum can be readily modified by surface chemical functionalization to broaden its application in different areas [9]. The intrinsic modifications of the guar backbone are known to enhance stability and water-absorbing capacity [10]. Metal nanoparticles on the other hand are attractive tools in photothermal and cellular drug-delivery applications [11]. Among the different metal nanoparticles used, silver nanoparticles are gaining importance in biomedical applications due to their unique optical properties related to surface plasmon resonance and antimicrobial properties [12, 13]. Wound healing is often delayed by bacterial infestation at the wound site, and the inflammatory phase becomes chronic suppressing the regenerative phase. The antibacterial effect of silver was also applied earlier in antimicrobial material coatings and biological material decontamination [14, 15]. Silver nanoparticles impregnated in biocompatible polymeric matrix were therefore conceived for antimicrobial wound healing material. We intrinsically modified guar gum, galactomannan, into a new derivative guar gum alkylamine (GGAA). This GGAA was further loaded with silver nanoparticles (Agnp) and new nanocomposites (NAg-GGAA) developed for skin wound healing evaluations.

## 2. Materials and Methods

### 2.1. Materials

All reagents were of analytical grade. Silver nitrate, dextrose, ammonium hydroxide, benzoyl chloride, guar gum (GG), sodium hydroxide, acetone, hydrochloric acid, and dimethyl sulfoxide were procured from Merck (India). Solvents and epichlorohydrin were purchased from Spectrochem (India).

### 2.2. Preparation of Guar Gum Alkylamine

A three neck round bottom flask (500 mL), fitted with a condenser, a mechanical stirrer and a nitrogen gas inlet, was used for the reaction. Typically, 25 g of GG was reacted with epichlorohydrin in liquor ammonia for 2 h at 55 ± 5°C under stirring and nitrogen purging [10]. The resultant guar gum alkylamine (GGAA) was filtered, washed in isopropanol, water and ethanol before being dried at 60°C in vacuum desiccators. The yield average recorded after six independent experiments was 19 g.

### 2.3. Preparation of Silver Nanocomposites In Situ

Silver nanoparticles stabilized in GGAA were prepared by reduction of ionic silver in alkaline (Na_2_CO_3_) dextrose. Twenty mM silver nitrate solution in 40 mL of 0.5% w/v GGAA was warmed (60°C), and a mixture of 2 mL, 25 mM dextrose in sodium carbonate (2 mL, 4 mM) was added for the reduction over a period of 10 min. The final straw yellow nanobio conjugate (NAg-GGAA) was separated upon addition of 50 mL acetone under stirring. The final products were nanoparticle embedded powders. The powdered product was washed thrice in 100 mL of 50% ethanol and subsequently dried in air oven at 60°C.

## 3. Characterization of the Silver Nanoparticles 

### 3.1. UV-Vis Spectroscopy

Plasmon response was recorded in a UV-vis spectrophotometer (Shimadzu UV-2550). The samples were dissolved in HPLC water and were scanned at slow speed at a resolution of 0.5 nm.

### 3.2. X-Ray Diffraction Analysis

XRD was used to determine material crystallinity and the characteristics of the silver nanoparticle in the new nanobio material. Samples were exposed to a generator voltage of 45 KV at 25 mA using Cu K*α* radiation in a PANalytical model PW 3040/60 X'Pert Pro defractometer. The diffraction angle (2*θ*) range of observation was 0–100° in a continuous scan mode at a scan rate of 0.5°/min at constant temperature of 22 ± 1°C. 

### 3.3. Scanning Electron Microscopy (SEM) with Energy Dispersive X-Ray Device (EDX)

The surface morphology of samples was examined in a scanning electron microscope (Philips, XL30) equipped with an energy dispersive X-ray device (EDX) attachment. SEM was operated at 10 kV after spattering the dried samples with gold. EDX spectrum was recorded from the samples by focusing the electron beam at specific regions of the nanomaterial.

### 3.4. Wound Healing Experiments

#### 3.4.1. Experimental Animals

 Male Wistar rats 150–200 g were used in the wound healing experiments. Animals were acclimatized for 7 days prior to the start of experiments in the laboratory housing conditions of 26°C  ±  2°C, 60–70% RH, and 12-hour light and dark cycle. All the experimental procedures and protocols used in this study were prepared as per the OECD Guidelines and Gaitonde Committee Guidelines and were approved by the Institutional Animal Ethical Committee (IAEC, registration no. 506/05/b/CPSCA). Animals were maintained with pellet foods available from Lipton India Pvt. Ltd, and water was allowed *ad libitum*.

#### 3.4.2. Punch Wound Model

Twenty-four hours before the beginning of wound healing experiments, the dorsal skin of the rats was shaved. The next day, full thickness wounds of 8 mm diameter were inflicted on the back side of each rat using a special type of sterile circular blade, Acu-Punch (Acuderm Inc., FL, USA), under light ether anesthesia. The animals were then divided into 4 groups (*n* = 6):

 Group I: normal paraffin treated (negative) control,
 Group II: guar gum alkylamine (GGAA) treated group,
 Group III: silver nano-GGAA (NAg-GGAA) treated group,
 Group IV: silver alginate cream (positive control).

Special care was taken for maintaining aseptic condition throughout the experiments. Test samples were dispersed in sterile water and were applied on the wounds topically at a fixed time each day. Silver alginate was also administered topically. 

#### 3.4.3. Determination of Wound Healing Rate

*Wound Closure Measurements*. The extent of wound closure was estimated by tracing the wound margin on a transparent graph paper in mm scale on days 0, 5, 7, and 10, respectively [16, 17]. The readings were indicative of the area of wound closure expressed in mm^2^ on respective days. The evaluated surface area was further used to determine the percentage of wound contraction on day 10. The following formula was used to determine % wound contraction:

(1) Original  wound  area  on⁡  day  0−wound  area  on⁡  day  10Original  wound  area  on⁡  day  0  ×100.

*Measurement of Wound Index*. Wound index was measured daily by an arbitrary scoring system (Table 1) [17]. 

#### 3.4.4. Measurement of Tensile Strength

 Tensile strength of wound indicates collagenesis of the healing process. The force required for opening the healed skin area is used as an indicator for completeness of healing. The tensile strength of newly repaired tissue of the wounds was measured using a tensiometer (M/S Excel Enterprises, Kolkata) after 10 days and were expressed in gm [17]. 

#### 3.4.5. Estimation of Biochemical Markers

Synthesis and deposition of proteins mark the initiation of the healing process after wounding. The protein and DNA contents reflect the process of cytokinesis during healing process [18]. The quality and quantity of protein deposited during the healing process significantly influence the strength of a scar. More than 50% of the protein in the scar tissue is made up of collagen, and collagenesis is essential for the healing process [19]. Hydroxyproline, the basic constituent of collagen, is an important marker of collagen synthesis [20]. 

For estimations of total protein and DNA content, the healed wound tissue, after complete healing (10th day), was excised using the 8 mm acupunch to avoid contamination with the host tissue. The designated tissues were then subjected to homogenization in 5% TCA and centrifuged [21]. The resultant pellet was washed with 10% TCA, resuspended in 5% TCA, and kept for 15 min in a water bath maintained at 90°C. The contents were centrifuged, and aliquots of DNA were derived from the supernatant for estimation by the method of Burton [22]. The precipitated proteins were suspended in 0.1 M Tris-HCl, pH 7.4, and the protein content was estimated by the method of Lowry et al. [23]. 

Tissues were dried in a hot-air oven at 60–70°C to get constant weight for measurement of hydroxyproline content. Weighed parts were digested in 6 N HCl at 130°C for 4 h in sealed tubes, and pH was adjusted to 7.0. Samples were further subjected to chloramines-T oxidation for 20 min, and the reaction was terminated by the addition of 0.4 M perchloric acid. Ehrlich reagent was used to develop color at 60°C, and the absorbance was measured at 557 nm using a spectrophotometer (Shimadzu UV-2550) [24]. 

#### 3.4.6. Histopathological Study of the Regenerated Tissues

Granular tissue samples from all groups collected upon sacrifice on day 10 were preserved immediately in 10% formalin solution and were evaluated by routine hematoxylin and eosin staining for histological criteria. The extent of reepithelialization, maturation, and organization of the epidermal squamous cells, the thickness of the granular cell layer, matrix organization, and cellular infiltration were observed. 

#### 3.4.7. Toxicological Evaluation of NAg-GGAA

*Primary Skin Irritation Experiment. *A primary skin irritation study was conducted with albino rabbit to determine the irritation potential of the NAg-GGAA. Each animal was treated with 500 *μ*L of NAg-GGAA and applied to the skin of one flank using a gauze patch. The patch was held in place with a semiocclusive bandage for 4 h, after which the patch was removed and the skin was cleaned of residual test drug. Skin reactions and irritation effects were assessed at approximately 1, 24, 48, and 72 h after the removal of the dressings. Adjacent areas of untreated skin from each animal served as controls. Erythema and edema were scored on a scale of 0–4, with 0 showing no effect and 4 representing severe erythema or edema.

### 3.5. Statistical Analysis

All the results presented here are shown as mean ± standard error of mean (SEM). The statistical significance was assessed using one-way analysis of variance (ANOVA) with *post hoc *pairwise comparisons between groups using the Bonferroni method. For all analyses, *P* < 0.05 was considered to be significant. Statistical analysis was performed using the computer statistical package SPSS/10.0 (SPSS, Chicago, IL, USA).

## 4. Results and Discussion

Wound management in the shortest possible time is of utmost importance, and the search for new biocompatible materials has led scientists to utilize the potential of polysaccharides derived from plants as wound management aids [5]. Polysaccharides or their derivatives can provide the ideal conditions for enhancing the wound healing process by actively participating in the wound healing process by facilitating cellular messaging and providing a hydrated surface. Silver is known for its antimicrobial activity since antiquity. Silver-containing drugs are also prevalent in markets and are applied to treat different kinds of wounds. However, many of these silver preparations cause cosmetic abnormality (argyria) and delayed wound healing for ionic silver reactions in biological fluids [25]. Silver nanoparticles associate strong antimicrobial potentials and are gaining importance due to their high surface area to volume ratio and unique physical and chemical properties [26]. We developed a newer preparation based on silver nanoparticles matrixed in hydropolymer guar gum for wound healing applications. 

### 4.1. Physicochemical Characterization of Guar Gum Alkylamine

Guar gum is a galactomannan in which one unit is substituted in every second galactose with a monomer mannose arranged in a chain. The new cationic modification is dependent upon incorporation of alkylamine groups on exposed hydroxyls. GGAA also forms stable films by itself or upon further hydrophobic substitution [27]. The new biomaterial properties were therefore dependent upon the number of head group substitutions on guar galactomannan backbone. 

#### 4.1.1. CHN Analysis and Degree of Substitution

Biopolymers were analyzed for C, H, N and percentage (w/w) composition and the degree of substitution. GGAA composition observations were C, 48.57%, N, 3.28%, H, 6.59% as compared to those of GG, C, 30.2%, and H, 4.79%. The degree of substitution for GGAA was calculated from nitrogen percentage estimations and was recorded as 0.457 (Table 2). 

### 4.2. Characterization of the Silver Nanoparticles

#### 4.2.1. UV-Vis Spectroscopy

**Figure 1:**
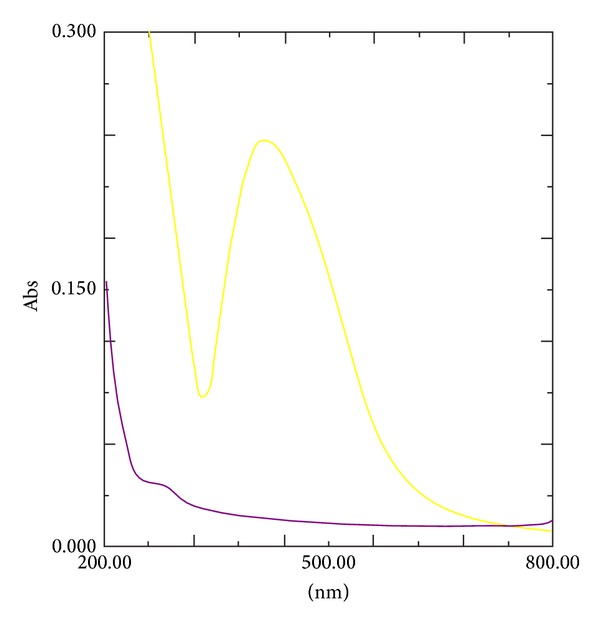
UV-spectra of GGAA and NAg-GGAA. Colour codes: GGAA (purple) and NAg-GGAA (yellow).

 The UV-Vis absorption spectra of the GGAA and NAg-GGAA were recorded in aqueous solution and are shown in Figure 1. The *λ* max of silver nanoparticles was observed at 415 nm. A remarkable broadening of peak at around 350 nm to 550 nm is indicative of polydispersity of the particles in the polymeric matrix. 

#### 4.2.2. X-Ray Diffraction Pattern

**Figure 2:**
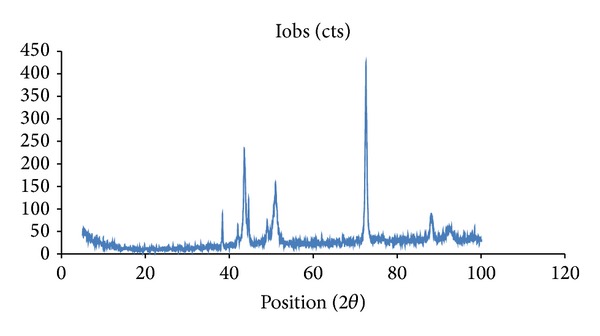
X-ray diffraction pattern of NAg-GGAA.

The XRD patterns of silver nanoparticles indicated a crystalline nature (Figure 2). Silver oxide peak at 2*θ* 38 degrees was negligible indicating purity of nanoparticles even after storage. This is likely due to GGAA protective capping of silver nanoparticles. Hundred-percent crystalline peak was observed at 2*θ*, 72 degree and corresponded with a near-spherical structure.

#### 4.2.3. Scanning Electron Microscopy

**Figure 3:**
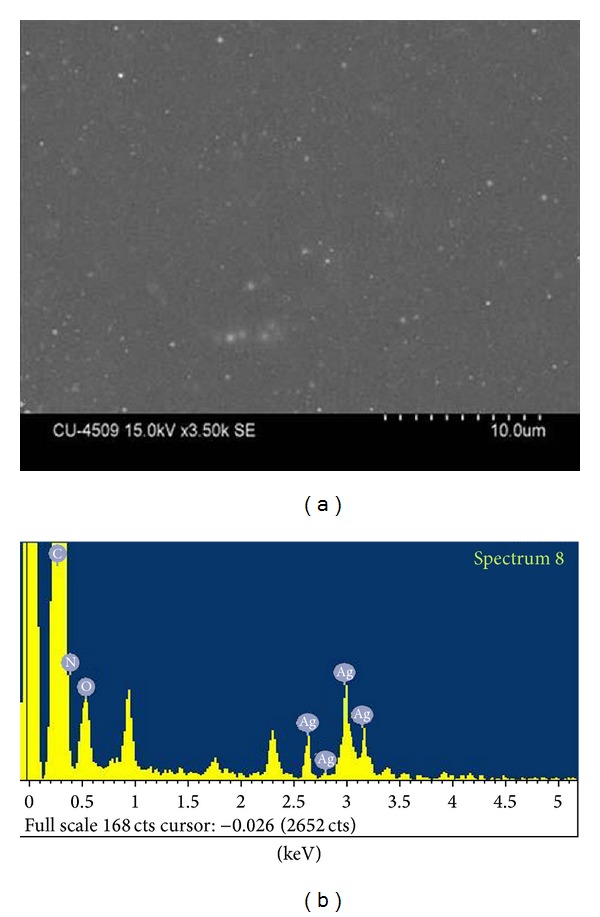
(a) SEM study of NAg-GGAA and (b) EDX spectra of NAg-GGAA showing the presence of silver peaks.

Scanning electron microscope pictures exhibit the surface morphology of biomaterials. Biopolymer embedded nanoparticles were observed in SEM studies and EDX studies (Figures 3(a) and 3(b)). The particles appeared dispersed uniformly in the polymeric matrix. The EDX spectra of the nanocomposite material showed the presence of characteristic silver peaks (Figure 3(b)) which confirms that reduction of Ag^+^ ions to nanocrystalline elemental silver.

### 4.3. Wound Healing Properties of NAg-GGAA 

#### 4.3.1. Effect on Wound Dimensions

**Figure 4:**
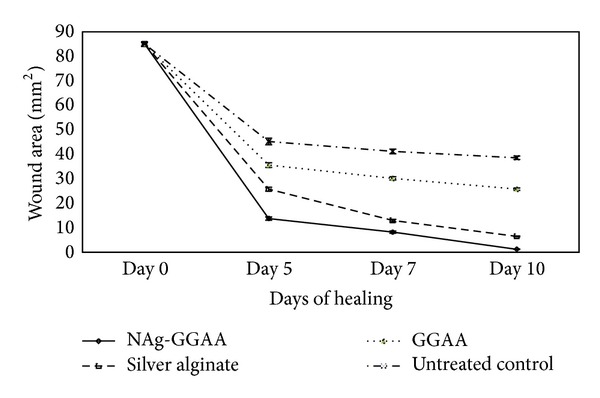
Area of wound closure in mm^2^ at 0th, 5th, 7th and 10th day. Results are mean ± SEM, *n* = 6 in each group, ^a^ *P* < 0.05 compared to control group, ^b^ *P* < 0.05 compared to GGAA treated group, ^c^ *P* < 0.05 compared to silver alginate group.

The extent of wound closure was estimated by tracing the wound margin on a transparent graph paper in mm scale on days 0, 5, 7, and 10, respectively. The readings were indicative of the area of wound closure expressed in mm^2^. The evaluated surface area was further used to determine the percentage of wound contraction on day 10 [28]. On the 5th day, the test group showed a significant reduction of wound size (13.8 mm^2^) in comparison to that of the untreated control (45.25 mm^2^). Complete closure was observed on day 10 (Figure 4). 

Similarly, on comparing the percentage of wound contraction, it was observed that the extent of healing was higher in NAg-GGAA group (~98%) in comparison to the silver alginate cream (~90%) with respect to day 0 wound size (*P* < 0.05). GGAA also showed about 69% reduction in wound size on day 10 compared to day 0 (Table 3). On day 10, however, only ~54% of the wound was closed in the untreated control group. 

#### 4.3.2. Effect on Wound Index

The mean wound indices on days 3, 5, 7, and 10 of each of the treatment groups were considered for evaluation. On day 10, the NAg-GGAA and silver alginate treated groups showed mean wound index of 1.35 and 1.56, respectively (*P* < 0.05), compared to untreated control. Mean wound indices of GGAA treated group also showed significant reduction (*P* < 0.05) as compared to control.

#### 4.3.3. Effect on Healing Period

On evaluating the number of days required for complete healing it was observed that the NAg-GGAA treated group showed complete healing on the 10th day. Silver alginate group took a healing time of 12 days for complete healing. Both are highly significant (*P* < 0.001) in comparison to the untreated control group which took 18 days to heal. Statistically significant faster healing was observed in NAg-GGAA treated group as compared to silver alginate group (*P* < 0.05). GGAA treated group also showed faster healing time (15 days, *P* < 0.05) as compared to control (Table 3). 

Open wounds are vulnerable to bacterial infections which interfere with the healing process and cause delayed healing. Silver nanoparticles exhibit enhanced rate of antimicrobial activity due to their unique physical properties such as high surface area to volume ratio and plasmon response. The antimicrobial activity of silver nanoparticles can be exerted by their interference with the cell membrane permeability causing degradation of the bacterial cell [29]. Additionally, silver nanoparticles can promote the rate of wound closure by promoting proliferation and migration of the keratinocytes at the wound site [30]. Also, silver nanoparticles can induce the differentiation of fibroblasts to myofibroblasts resulting in faster wound contraction.

Silver nanoparticles however tend to agglomerate, and they are short-lived in the circulation. As a result, silver nanoparticles are incorporated in suitable polymer matrix for the production of highly stable and uniformly distributed silver nanoparticles. The silver impregnated guar matrix provides a sustained release of silver while exerting its antimicrobial activity [31]. The polymeric matrix also provides a hydrated surface for wound healing. Thus, in most of the parameters the GGAA treated group showed positive result with respect to the control. 

#### 4.3.4. Tensile Strength in the Healing Wounds

A key parameter in healing involves regaining strength of the regenerated dermal matrix. Tensile strength reflects the quality and speed of tissue regeneration. Tensile strength is directly related to collagen content of wounds [32]. Collagen is the main element responsible for tissue integrity and provides a platform for reepithelialization and is essential for restoring skin functionality during injury. The mean tensile strength of the NAg-GGAA treated group (522 g) was significantly greater (*P* < 0.05) than that of the untreated control group (257 g) and silver alginate group (460 g) (Table 3). Higher tensile strength of GGAA-silver-nanocomposite treated wounds indicates better quality healing than the silver alginate treated wounds. The increase in tensile strength of treated wounds may be due to the increase in collagen concentration and twisting of the collagen fibers. The greater the tensile strength, the better the healing. The NAg-GGAA particles possibly modulate collagen deposition at the site of the wound during the healing process and promote a regulated differentiation of fibroblasts [33]. GGAA treated group also showed higher tensile strength (357 g) as compared to control (Table 3). 

#### 4.3.5. Changes in the Biochemical Markers of the Healed Wounds

DNA and total protein content are indicative markers of cell growth after tissue injury [34, 35]. The hydroxyproline content, DNA, and total protein contents of granulation tissues after complete healing are given in Table 4. The mean hydroxyproline content was found to be significantly higher in all treatment groups when compared to the untreated control (*P* < 0.01). Thus, high levels of hydroxyproline indicate higher collagen production which is essential for the healing process [17]. The DNA content in wounds treated with NAg-GGAA and silver alginate treated groups was significantly higher than in the untreated control group (*P* < 0.001). However, DNA content of wound tissue of NAg-GGAA treated group was statistically higher (*P* < 0.05) than that of silver alginate treated group. Similar phenomenon was noted in case of total protein content of wound tissues where NAg-GGAA and silver alginate treated groups showed higher protein contents (23.9 and 21.3 mg/gm wet tissue, *P* < 0.001) when compared to the untreated (paraffin treated) control group (15.1 mg/gm wet tissue) but total protein content of NAg-GGAA treated wound tissue was statistically higher (*P* < 0.05) than that of silver alginate treated group. Increased DNA content may be related to the upregulation of zinc metabolism which leads to enhanced production of RNA and DNA synthetases [36]. 

Thus, the higher levels of the major biochemical markers (compared to the untreated control) indicate cellular proliferation at the wound site and thereby faster healing of wound. Interestingly, the levels of all of the selected biochemical markers were statistically higher (*P* < 0.05) in NAg-GGAA treated group than that of silver alginate treated group indicating faster and quality healing (Table 4). 

#### 4.3.6. Histopathological Study of the Regenerated Tissues

**Figure 5:**
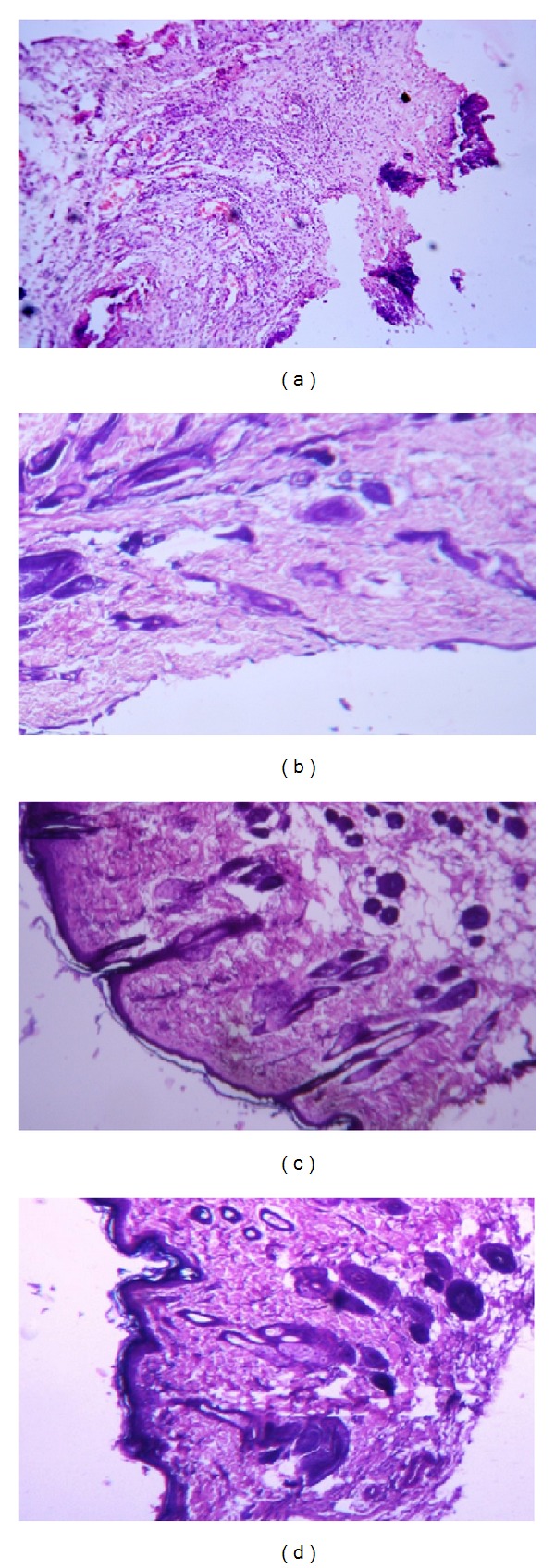
Hematoxylin and eosin stained granulation tissue at day 10. (a) Untreated control group, (b) GGAA treated group, (c) silver alginate cream with thin layer of epithelialization, and (d) NAg-GGAA treated group showing well organized granulation tissue and epithelialization (magnification 40x).

Hematoxylin and eosin (H&E) stained sections of granulations tissue on day 10 of the untreated control animals (Figure 5(a)) showed the presence of acute inflammatory cells and very few blood vessels which were prominent and dilated. It also showed lesser epithelialization and lesser collagen formation. In contrast, a well organized granulation tissue was observed in the NAg-GGAA treated group (Figure 5(d)). New blood vessel formation, epithelialization, and increase in fibroblast cells were observed in the silver nano-GGAA treated group. The silver alginate treated group showed granular tissue regeneration and new blood vessel formation but poor epithelialization (Figure 5(c)). The GGAA (Figure 5(b)) showed regenerative changes with poor epithelial layer formation. These results indicate that the tissue architecture was well advanced on day 10 in the Ag-nano-GGAA treated group compared to the other groups. Further, the cosmetic appearance was much improved in the NAg-GGAA treated group.

#### 4.3.7. Dermal Toxicity Study

No irritation was observed following the 4 h dermal exposure of NAg-GGAA over the skin of the test animals.

## 5. Conclusion

We have developed silver nanocomposites embedded in cationic guar gum polymeric matrix. The new nanobiocomposite was further evaluated for wound healing application in rat punch wound model. The new GGAA matrix provided stabilization of silver nanoparticles. The embedded spherical silver nanoparticles were well dispersed in the polymeric matrix. Wound healing experiments with NAg-GGAA have demonstrated the superior wound healing efficacy of the new nanocomposite as compared to that of silver alginate cream in parallel runs. The nanobiocomposite promote wound healing by modulation of collagen deposition and regulation of keratinocytes and support the essential re-epithelialization process.

## Figures and Tables

**Table 1:** Arbitrary scoring system for the measurement of wound index.

Gross changes	Wound index
Complete healing of wounds	1
Delayed but healthy healing	2
No initiation of healing, but the environment is healthy	3
Formation of pus: evidence of necrosis	4

Total	10

**Table 2:** Physicochemical characterization of guar gum and its newer derivatives.

Percentage composition CHN analysis in combustion technique									Degree of substitution
Compound no.	Code	Compound name	Calculated %			Observed %			
			C	H	N	C	H	N	
I	GG	Guar gum	32.9	4.57	—	30.2	4.79	—	—
II	GGAA	Guar gum alkylamine	45.56	5.60	2.53	48.57	6.59	3.28	0.457

**Table 3:** Changes in physical characteristics of wounds in different treatment groups.

Treatment groups	Percentage wound contraction on the 10th day	Wound index	Healing period (days)	Tensile strength (g)
Untreated control	54.58 ± 3.45	2.69 ± 0.08	18.83 ± 0.48	257.2 ± 10.73
GGAA treated	69.64 ± 4.86^a^	1.94 ± 0.06^a^	15.50 ± 0.43^a^	356.8 ± 9.88^a^
NAg-GGAA treated	98.52 ± 7.54^a,b,c^	1.35 ± 0.05^a,b^	9.83 ± 0.31^a,b,c^	522.5 ± 12.9^a,b, c^
Silver alginate	89.15 ± 6.96^a,b^	1.56 ± 0.05^a,b^	12.5 ± 0.43^a,b^	460.5 ± 11.66^a,b^

Values were mean ± SEM, *n* = 6 in each group. ^a^ *P* < 0.05 compared to control group, ^b^ *P* < 0.05 compared to GGAA treated group, ^c^ *P* < 0.05 compared to silver alginate group.

**Table 4:** Changes in biochemical parameters of wound tissues in different treatment groups.

Treatment groups	DNA (mg/g tissue)	Total protein (mg/g tissue)	Hydroxyproline (*µ*g/mg tissue)
Untreated control	1.02 ± 0.06	15.13 ± 0.18	0.81 ± 0.02
GGAA treated	1.30 ± 0.03^a^	16.98 ± 0.18^a^	1.30 ± 0.03^a^
NAg-GGAA treated	2.36 ± 0.04^a,b,c^	23.91 ± 0.19^a,b,c^	2.36 ± 0.04^a,b,c^
Silver alginate	2.04 ± 0.09^a,b^	21.27 ± 0.37^a,b^	2.04 ± 0.09^a,b^

Values were mean ± SEM, *n* = 8 in each group. ^a^ *P* < 0.05 compared to control group, ^b^ *P* < 0.05 compared to GGAA treated group, ^c^ *P* < 0.05 compared to silver alginate group.
